# Novel postzygotic *RASA1* mutation in a patient with Parkes Weber syndrome: A case report and literature review

**DOI:** 10.1002/ccr3.9543

**Published:** 2024-11-03

**Authors:** Robin A. Pilz, Dariush Skowronek, Tamara Ehresmann, Ute Felbor, Matthias Rath

**Affiliations:** ^1^ Department of Human Genetics University Medicine Greifswald, and Interfaculty Institute of Genetics and Functional Genomics, University of Greifswald Greifswald Germany; ^2^ Limbach Genetics GmbH MVZ Humangenetik Frankfurt Germany; ^3^ Institute for Molecular Medicine, MSH Medical School Hamburg Hamburg Germany

**Keywords:** capillary malformation, de novo variant, mosaicisms, Parkes Weber syndrome, *RASA1*, segmental overgrowth

## Abstract

**Key Clinical Message:**

Not only germline but also postzygotic mutations in the *RASA1* or *EPHB4* genes can lead to capillary malformation‐arteriovenous malformation (CM‐AVM) syndrome. As it is not always possible to clinically distinguish between constitutional variants and postzygotic mosaicism, a sufficiently high sequencing depth must be used in genetic diagnostics to detect both.

**Abstract:**

Capillary malformation‐arteriovenous malformation (CM‐AVM) syndrome, with or without Parkes Weber syndrome, is a rare autosomal dominant disease caused by pathogenic *RASA1* or *EPHB4* variants. Up to 80% of CM‐AVM cases have an affected parent. Gene panel sequencing was performed for a 4‐year‐old girl with multiple CMs, two capillary stains on the left leg, and associated overgrowth of the second toe. We also reviewed published cases with mosaic *RASA1* and *EPHB4* mutations. A mosaic *RASA1* loss‐of‐function mutation was detected with a variant allele frequency (VAF) of 20% in the blood and oral epithelial cells of the index patient. The literature review illustrates that the severity of the clinical phenotype does not correlate with the VAF. We also identified a germline nonsense variant in the patient's *TEK* gene. However, inactivating *TEK* variants do not cause a vascular phenotype but can confer an increased risk for primary congenital glaucoma with variable expressivity. The case presented here illustrates that the choice of the sequencing depth of a diagnostic next‐generation sequencing test for CM‐AVM patients should always take mosaicism into account and that a good knowledge of the sequenced genes and associated disease mechanisms is necessary for adequate genetic counseling.

## Graphical abstract

Capillary malformation‐arteriovenous malformation (CM‐AVM) syndrome, with or without Parkes Weber syndrome, is a rare autosomal dominant disease. We here present a Parkes Weber syndrome patient with a negative family history and a mosaic *RASA1* loss‐of‐function mutation detected with a variant allele frequency of 20% in her blood and oral epithelial cells. Our case report and literature review demonstrate the importance of genetic mosaicism in CM‐AVM and further illustrate the increasing complexity of genetic diagnostics.

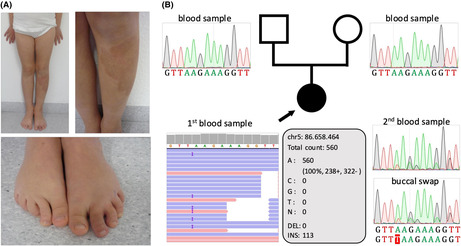

## 1 INTRODUCTION

Capillary malformation‐arteriovenous malformation (CM‐AVM) syndrome is an autosomal dominant vascular disorder caused by loss‐of‐function variants in either the *RASA1* (CM‐AVM1; OMIM: 608354)^1^ or *EPHB4* gene (CM‐AVM2, OMIM: 618196).^2^ The prevalence is estimated to be about 1:20,000 and 1:12,000, respectively.^3^ In venous endothelial cells, the Ras GTPase‐activating protein 1 (RASA1, p120 RasGAP) and the Ephrin type‐B receptor 4 (EPHB4) act together as negative regulators of the RAS‐MAPK‐ERK1/2 and PI3K‐AKT‐mTORC1 pathways.^2^, ^4^ Thus, both proteins are essential for organized vascular development and the formation of regular capillary beds. However, *EPHB4* mutations have also been described in other diseases, for example, in venous valvular aplasia, lymphatic‐related hydrops fetalis, or adolescent‐onset primary lymphedema.^5^, ^6^, ^7^

CM‐AVM patients with pathogenic *RASA1* or *EPHB4* variants usually present with multiple small (1–2 cm), pinkish‐to‐red, round‐to‐oval capillary malformations (CM).^8^ These slow‐flow lesions are mostly found on the face or extremities and sometimes surrounded by a pale halo.^9^ In up to every third CM‐AVM1 patient, additional arteriovenous malformations (AVM) and arteriovenous fistulas (AVF), which both belong to the group of fast‐flow lesions, or a Parkes Weber syndrome (PWS) can be found.^8^ The latter is characterized by the simultaneous occurrence of a cutaneous capillary stain, multiple micro‐AVFs, and hypertrophy of the affected limb.^3^, ^9^ CM‐AVM2 patients may present not only with additional fast‐flow vascular malformations but also telangiectasias, Bier spots, and epistaxis.^2^ The penetrance of CM‐AVM1 and CM‐AVM2 is almost 99% and 93%, respectively.^2^, ^9^ Next‐generation sequencing (NGS) gene panel tests are now widely used to detect a pathogenic germline variant and thus confirm a hereditary CM‐AVM disease or address differential diagnoses in clinically less specific cases. However, postzygotic de novo mutations in *RASA1* and *EPHB4* must also be considered to maximize the clinical sensitivity of the genetic test.^3^, ^10^, ^11^, ^12^ We here present the case of a young PWS patient with a postzygotic de novo variant in the *RASA1* gene and provide a literature review on mosaic variants in CM‐AVM and PWS patients.

## 2 CASE HISTORY

**FIGURE 1:**
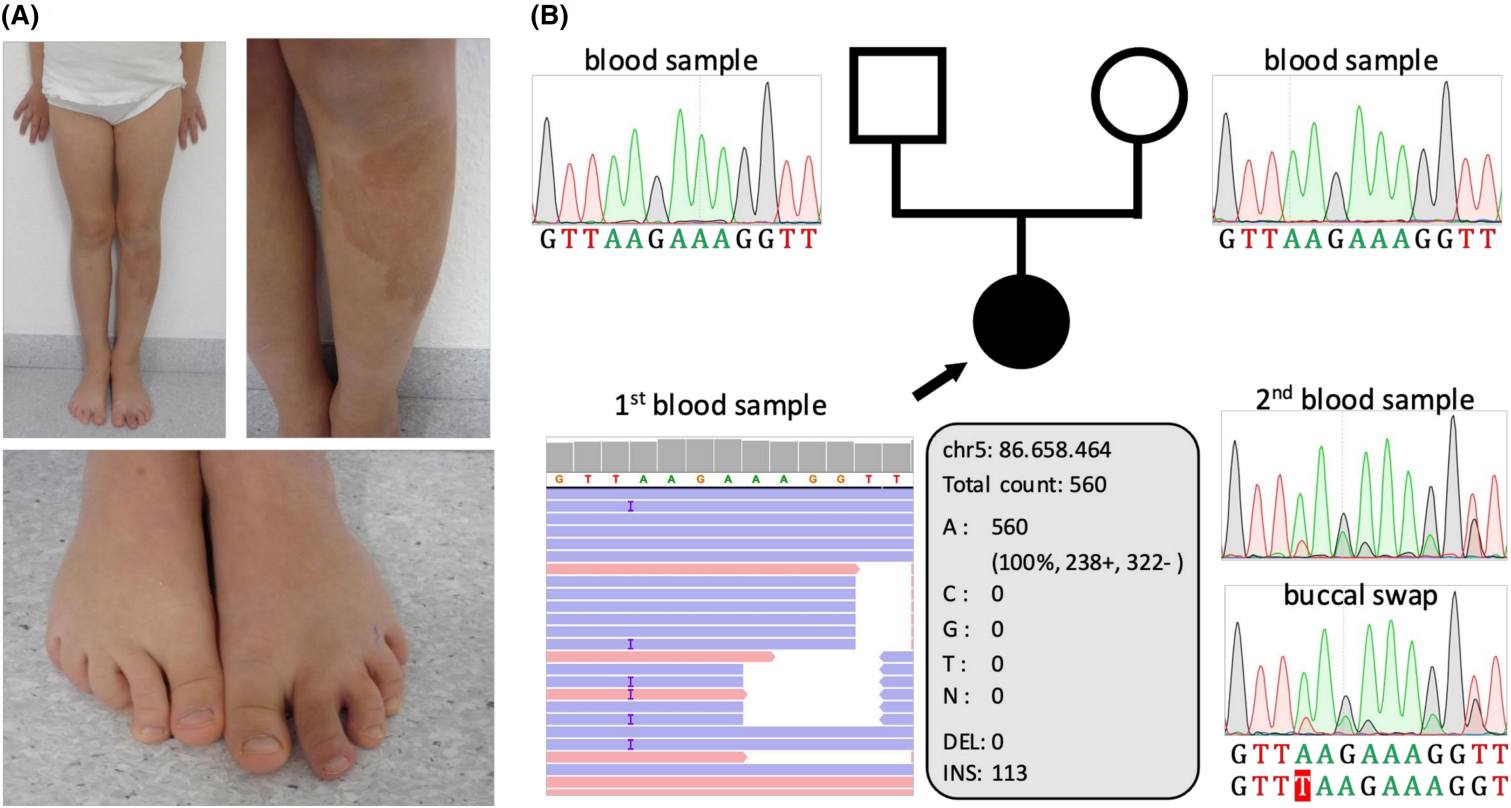
Index proband with a Parkes Weber syndrome and a postzygotic *RASA1* variant. (A) Large capillary stain on the left lower limb with associated segmental overgrowth, especially of the second toe. (B) NGS gene panel analysis and Sanger sequencing detected a *RASA1* variant [c.1428dup; p.(Lys477*)] with a variant allele frequency of approx. 20% in two independent blood samples and an additional buccal mucosa sample of the patient. The pathogenic *RASA1* variant was not detected in the blood samples of the parents.

The female index case was referred to NGS gene panel analysis for vascular malformations at the age of 4 ^10^/_12_ years. Growth and development were normal. Clinical examination revealed multiple CMs, a large capillary stain on her lower left leg, and another on the sole of her left foot associated with overgrowth of the second toe (Figure 1A). The parents reported that the two vascular lesions on the lower extremity were already present at birth. Micro‐AVFs were suspected, but no diagnostic imaging had been performed for the index case. No vascular phenotype was reported for her parents, her younger brother, and other relatives.

## 3 GENETIC ANALYSES

Genomic DNA samples were isolated from peripheral blood lymphocytes of the index proband and her parents with the NucleoSpin Blood L kit (Machery‐Nagel, Düren, Germany) or buccal mucosa of the index proband using the Gentra Puregene Buccal Cell Kit (Qiagen, Hilden, Germany).

All coding exons (±20 bp) of the *RASA1* (OMIM: 139150), *EPHB4* (OMIM: 600011), *TEK* (OMIM: 600221), and *GLMN* (OMIM: 601749) genes were defined as target regions for NGS gene panel analysis. An Agilent SureSelect^QXT^ custom enrichment kit (Panel ID: 3152261, Agilent Technologies, Santa Clara, CA, USA) was used for target enrichment and library preparation according to the manufacturer's instructions. Indexed libraries were pooled and sequenced on a MiSeq instrument (Illumina, San Diego, CA, USA) as 2 × 150 bp paired‐read runs. The MiSeq Reporter Software (Illumina) was used for demultiplexing and FASTQ file generation. Mapping, alignment, and variant calling were performed with the SeqNext module of the Sequence Pilot software (JSI Medical Systems, Ettenheim, Germany). The SeqNext module was also used for CNV (copy number variant) analyses.

For the verification of the identified variant in an independently obtained blood and a buccal swap sample of the index proband, the specific region of the *RASA1* gene was first amplified by PCR and then analyzed by Sanger sequencing on an ABI 3130xl (Applied Biosystems, Waltham, MA, USA) using the BigDye Terminator Cycle Sequencing v3.1 kit (Applied Biosystems). Primer sequences are available upon request.

## 4 CONCLUSIONS AND RESULTS

Sequencing of a blood sample from the proband revealed a 1‐base pair duplication in exon 10 of the *RASA1* gene (ENST00000274376.11): c.1428dup; p.(Lys477*). However, the identified duplication, which introduces a premature stop codon in the pleckstrin homology (PH) domain of the Ras GTPase‐activating protein 1, was only present in 20% of all reads (Figure 1B). The Sanger sequencing results for a second independent blood sample and a buccal mucosa sample of the index proband strengthened the hypothesis that it is a postzygotic variant. Tissue samples of the vascular lesions were not available. Consistent with the assumption of a postzygotic origin, the variant was not detected in the blood samples of both parents (Figure 1B). The identified loss‐of‐function variant in *RASA1* was not found in mutation databases (e.g., ClinVar, Global Variome shared LOVD *RASA1*/“Vascular anomalies and lymphedema” gene variant database), in the literature, or the Genome Aggregation Database (gnomAD v2.1.1 data set). Bioinformatic evaluation using several in silico prediction tools (Ensembl VEP, MutationTaster2021, NMDEscPredictor, SIFT Indel, MutPred‐LOF) supports the pathogenicity of the variant which is predicted to result in nonsense‐mediated decay (NMD). Loss of protein expression for *RASA1* null variants has been shown in the literature.^13^ Using the guidelines of the American College of Medical Genetics and Genomics and the Association for Molecular Pathology,^14^ the postzygotic *RASA1* variant was classified as pathogenic for CM‐AVM (criteria: PVS1, PM2, PM6). Based on the genetically confirmed diagnosis of CM‐AVM with Parkes Weber syndrome, we recommended referral to a specialized center for vascular malformations and further clinical investigations for AVM‐ or AVF‐associated complications.

Interestingly, a second loss‐of‐function variant was identified in the *TEK* gene (ENST00000380036.10) of the index proband: c.2620G > T; p.(Glu874*). This variant causing a stop codon in the kinase domain of the TEK protein was inherited from the proband's 40‐year‐old mother and was listed in the gnomAD browser (v2.1.1) only once in a heterozygous state. Bioinformatic tools (Ensembl VEP, MutationTaster2021, BayesDel, CADD, MutPred‐LOF) predict the variant to be deleterious. Loss‐of‐function variants in the *TEK* gene have been described as disease‐causing for primary congenital glaucoma (PCG) and were validated experimentally.^15^ However, several *TEK* mutation carriers without a typical congenital or early‐onset glaucoma phenotype have also been reported.^15^ In line with this observation, our index patient and her mother also showed no abnormalities in ophthalmologic examinations.

## 5 DISCUSSION

Tests used in human genetic diagnostics must always undergo extensive validation to ensure their ability to detect pathogenic germline variants with high sensitivity. However, the case of a PWS patient with a postzygotic *RASA1* variant that we present here illustrates once more that genetic mosaicism, which is often outside the focus of genetic germline analyses, must always be kept in mind. Genetic mosaicism describes “the presence of two or more cell lineages with different genotypes arising from a single zygote in a single individual”.^16^ It is caused by variants that are not inherited but acquired after formation of the zygote.^17^ Various monogenic diseases, for example, neurocutaneous or neurodevelopmental disorders, can be caused by pathogenic variants in a mosaic state.^18^, ^19^ In these cases, the detection of a postzygotic variant is not only essential for confirming the diagnosis but also for genetic counseling.

Although patients with a postzygotic variant are often thought to have a milder phenotype compared to patients with an inherited germline variant,^20^ Table 1 does not reveal a correlation between the phenotype and the variant allele read frequency in the blood of CM‐AVM patients with a postzygotic *RASA1* or *EPHB4* variant. Thus, the detection of a postzygotic mutation in our PWS patient could not be used to predict the clinical course. Nevertheless, it was a great relief for the parents to know that there is no increased risk for further children to develop a PWS phenotype. However, it is possible that the index proband herself will pass on the pathogenic variant to future children since the patient's germ cells may carry the 1‐base pair duplication within the *RASA1* gene.

**TABLE 1:** Mosaic *RASA1* and *EPHB4* variants detected in blood samples of CM‐AVM patients with or without PWS previously described in the literature or in this study.

References	Patients	Gene	Variant	VAF	Phenotype
^10^	1	*RASA1*	c.1248T > A; p.(Tyr416*)	7% (blood, cov = 611x) 19% (CM, cov = 185x)	CM + AVM + overgrowth
	2	*RASA1*	c.2131C > T; p.(Arg711*)	21.5% (blood, cov = 274x)	CM + AVM
^11^	3	*RASA1*	c.1879A > T; p.(Lys627*)	25.3%/35.7% (blood, cov = 649x/28x)	CM + AVF/AVM
	4	*RASA1*	c.2035C > T; p.(Arg679*)	2.7%/3.1% (blood, cov = 111x/2011x) 13.6% (AVM, cov = 3407x)	CM + Bier spots + telangiectasias + AVM
	5	*RASA1*	c.1192C > T; p.(Lys398*)	8.5% (blood, cov = 1189x)	CM + AVF + overgrowth
	6	*RASA1*	c.2707C > T; p.(Arg903*)	6.1% (blood, cov = 964x) 6.9% (CM, cov = 305x)	CM
^26^	7	*EPHB4*	c.2450G > A; p.(Gly817Glu)	18.6% (blood, cov = 851x)	CM + telangiectasias + epistaxis + Bier spots
^12^	8	*RASA1*	c.3069del; p.(Lys1025Serfs*5)	8% (blood)	CM + AVF + overgrowth
This study	9	*RASA1*	c.1428dup; p.(Lys477*)	20.2% (blood, cov = 560x)	CM + AVF(?) + overgrowth

*Note*: Somatic *RASA1* mutations only detected in tissue samples of CM‐AVM patients and mosaic *EPHB4* variants detected in patients with venous valve aplasia are not included in this table.

Abbreviations: AVF, arteriovenous fistula; AVF(?), suspected arteriovenous fistula; AVM, arteriovenous malformation; CM, capillary malformations; cov, sequencing depth.

Depending on when a genetic variant occurs in postzygotic development and how many cells or tissues are affected, different types of mosaicism have been described. One type is postnatal somatic mosaicism^21^ that can usually only be detected in affected tissue but not in blood. Somatic mutations have been identified several times in vascular lesions of CM‐AVM patients.^22^, ^23^ However, tissue samples are often only available in a research context. In contrast to postnatal somatic mosaicism, early postzygotic variants are present in all tissues and can also be detected in blood. The presence of the *RASA1* variant in mesoderm‐derived white blood cells and ectoderm‐derived oral epithelial cells^24^ of the index proband suggests that the variant has arisen in very early mitotic cell divisions after fertilization. Yet, a minimum sequencing depth of 30×, as often used in germline assays, is not always sensitive enough to detect mosaic variants. Therefore, a minimum sequencing depth of at least 100× is recommended.^25^ Although higher sequencing depths will result in higher expenses, genetic testing for CM‐AVM patients should always be validated for germline and early postzygotic variants.

A second lesson from our case report is that molecular and clinical geneticists have to have a good knowledge of the genes they are analyzing and the associated pathomechanisms to avoid misdiagnosis. The index proband and her mother are carriers of a nonsense variant in *TEK*. It is essential to know that activating but not inactivating *TEK* variants can lead to multiple cutaneous and mucosal venous malformations (VMCM). *TEK* variants with loss‐of‐function are instead associated with primary congenital glaucoma with incomplete penetrance and variable expressivity.^15^ Maintaining this expertise even in large panel, exome, and genome sequencing analyses may not always be easy.

In summary, our case report and literature review demonstrate the importance of genetic mosaicism in CM‐AVM and further illustrate the increasing complexity of genetic diagnostics requiring extensive evaluation of the identified variants in order to provide the best possible information to advice‐seeking patients and their families.

## AUTHOR CONTRIBUTIONS

**Robin A. Pilz:** Data curation; formal analysis; investigation; visualization; writing – original draft; writing – review and editing. **Dariush Skowronek:** Data curation; formal analysis; writing – review and editing. **Tamara Ehresmann:** Investigation; resources; writing – review and editing. **Ute Felbor:** Data curation; supervision; writing – original draft; writing – review and editing. **Matthias Rath:** Data curation; formal analysis; investigation; supervision; visualization; writing – review and editing.

## FUNDING INFORMATION

This research did not receive any specific grant from funding agencies in the public, commercial, or non‐for‐profit sectors.

## CONFLICT OF INTEREST STATEMENT

The authors declare that they have no competing interests.

## ETHICS APPROVAL

The study protocol was approved by the local ethics committee of the University Medicine Greifswald (BB 047/14a).

## CONSENT

Written informed consent was obtained from the patient to publish this report in accordance with the journal's patient consent policy.

## Data Availability

The data supporting the results of this study are available on reasonable request from the corresponding author. The NGS data are not publicly available due to data protection restrictions.
